# The impact of pyroclastic density currents duration on humans: the case of the AD 79 eruption of Vesuvius

**DOI:** 10.1038/s41598-021-84456-7

**Published:** 2021-03-02

**Authors:** Pierfrancesco Dellino, Fabio Dioguardi, Roberto Isaia, Roberto Sulpizio, Daniela Mele

**Affiliations:** 1 Dipartimento Di Scienze Della Terra E Geoambientali, Università Di Bari, Bari, Italy; 2 British Geological Survey, The Lyell Centre, Edinburgh, UK; 3 Osservatorio Vesuviano, Istituto Nazionale Di Geofisica E Vulcanologia, Sezione Di Napoli, Napoli, Italy

**Keywords:** Natural hazards, Solid Earth sciences

## Abstract

Pyroclastic density currents are ground hugging gas-particle flows that originate from the collapse of an eruption column or lava dome. They move away from the volcano at high speed, causing devastation. The impact is generally associated with flow dynamic pressure and temperature. Little emphasis has yet been given to flow duration, although it is emerging that the survival of people engulfed in a current strongly depends on the exposure time. The AD 79 event of Somma-Vesuvius is used here to demonstrate the impact of pyroclastic density currents on humans during an historical eruption. At Herculaneum, at the foot of the volcano, the temperature and strength of the flow were so high that survival was impossible. At Pompeii, in the distal area, we use a new model indicating that the current had low strength and low temperature, which is confirmed by the absence of signs of trauma on corpses. Under such conditions, survival should have been possible if the current lasted a few minutes or less. Instead, our calculations demonstrate a flow duration of 17 min, long enough to make lethal the breathing of ash suspended in the current. We conclude that in distal areas where the mechanical and thermal effects of a pyroclastic density currents are diminished, flow duration is the key for survival.

## Introduction

The impact of pyroclastic density currents (PDCs) is generally attributed to the combination of flow temperature and dynamic pressure^1–3^. The latter is expressed by the dynamic pressure,

1 $$ P_{dyn} = \frac{1}{2}\rho_{mix} U^{2} $$

that represents the lateral force per unit area acting on buildings and living bodies*,* where

2 $$ \rho_{mix} = \rho_{s} C + \rho_{g} \left( {1 - C} \right) $$

is the gas-particle mixture density, *ρ*_*s*_ and *ρ*_*g*_ are particle and gas density, *C* is particle volumetric concentration and *U* is current velocity. A complete symbol list is found in Table 1.

**Table 1:** List of symbols, with description and physical dimension.

Symbol	Description	Dimension
*A*_*r*_	Aggradation Rate	m s^−1^
*C*_*0*_	Reference particle concentration (0.7)	–
*C*	Particle volumetric concentration	–
*C*_*tot*_	Total particle volumetric concentration	–
*C*_*d*_	Particle drag coefficient	–
*C*_*sf*_	Depth-averaged concentration in the basal shear flow	–
*C*_*pa*_	Air specific heat	J kg^−1^ °C^−1^
*C*_*pg*_	Gas specific heat	J kg^−1^ °C^−1^
*C*_*ps*_	Solid specific heat	J kg^−1^ °C^−1^
*d*	particle size	mm
*g*	Gravity acceleration (9.81)	m s^−2^
*g*′	Reduced gravity	m s^−2^
*H*	Total flow thickness	m
*H*_*dep*_	Deposit thickness	m
*H*_*sf*_	Shear flow height	m
*k*	Von Karman constant (0.4)	–
*k*_*s*_	substrate roughness	m
*P*_*dyn*_	Dynamic pressure	k Pa
*P*_*n*_	Particle Rouse number	–
*P*_n_*	Normalized Rouse number	–
*P*_*navg*_	Average Rouse number of the solid material	–
*P*_*ni*_	Rouse number of the ith particle-size class	–
*P*_*nsusp*_	Rouse number at maximum suspension capacity	–
*Ri*_*0*_	Richardson number	–
*S*_*r*_	Sedimentation rate	kg m^−2^ s^−1^
*t*	Aggradation time	s
*T*_*a*_	air temperature	°C
*T*_*g*_	Gas temperature	°C
*T*_*mix*_	Temperature of mixture	°C
*T*_*s*_	solid temperature	°C
u_*_	Flow shear velocity	m s^−1^
*U*	Current velocity	m s^−1^
*w*_*t*_	Particle terminal velocity	m s^−1^
*w*_*ti*_	Terminal velocity of the ith particle-size class	m s^−1^
*y*	Flow vertical coordinate	m
*y*_*0*_	Basal lamina thickness	m
*α*	Slope angle	Deg
*ϕ*_*i*_	Weight fraction of the i_th_ size class	Weight%
*ρ*_*a*_	Atmospheric density	kg m^−3^
*ρ*_*dep*_	Deposit density	kg m^−3^
*ρ*_*g*_	Gas density	kg m^−3^
*ρ*_*mix*_	Density of the gas–particle mixture	kg m^−3^
*ρ*_*s*_	Particle density	kg m^−3^
*ρ*_*sf*_	Shear flow density	kg m^−3^
*ρ*_*si*_	Density of the ith particle-size class	kg m^−3^
*τ*	Shear-driving stress of shear flow	Pa

Engineering investigations^1,4,5^ show that dynamic pressures higher than 5 kPa produce significant damage, while pressures under 1 kPa have minimal to no consequence on structures or infrastructures. Particle volumetric concentration represents an important parameter too because dynamic pressure is proportional to it. Currents moving in the vicinity of a volcano can have a high concentration of hot magmatic particles that confer high temperature and high dynamic pressure to the flow. This can cause burning of buildings, breaking of windows and toppling of walls, which make survival impossible^6^.

Concerning effects on humans, it is emerging that even in areas far from a volcano, where particle concentration, temperature and dynamic pressure strongly decrease, people engulfed in the flow have “high probability of receiving fatal skin burns and inhalation injury of the upper and lower respiratory tract, unless the duration is very brief”^7^. The presence of fine-ash particles suspended in air for a long time, even in very small amounts, can be very harmful to human health, and represents one major cause of injury^2^. Exposure to pure hot air at 200–250 °C can be survived for 2–5 minutes^8^, but the presence of inhalable hot fine ash drastically reduces survival times. The exposure time therefore plays a major role in determining the impact of PDCs on human beings, but, until now, it has not been quantified^2,7^. We study here the famous AD 79 eruption of Somma-Vesuvius^9,10^ and we reconstruct, for the first time, also the effect of flow duration on humans.

### The 79 AD eruption of Vesuvius and associated deposits

**Figure 1:**
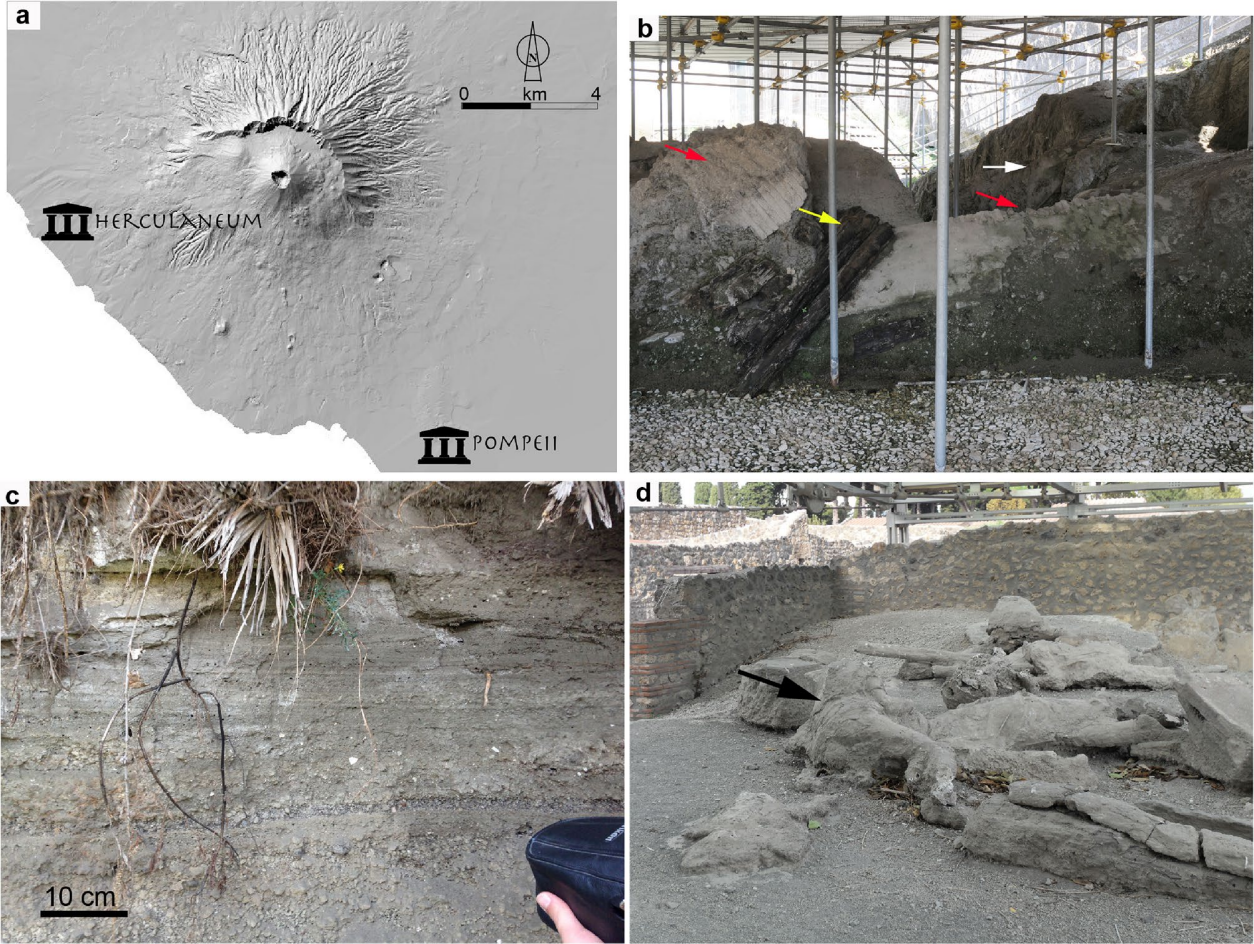
The PDC deposits of the AD 79 Vesuvius eruption. (**a**)—Map showing Herculaneum and Pompeii locations (courtesy of Osservatorio Vesuviano); (**b**)—Herculaneum: the white arrow shows the massive bed formed by the concentrated current that caused charring of woods (yellow arrow) and toppling of walls (red arrows); (**c**)—Pompeii: the stratified layer with tractional structures that was formed by the stacking up of laminae during suspension sedimentation from the dilute PDC, is shown; (**d**)—Pompeii: some corpses, embedded in the ash layer, which show intact bodies and preserved dressings (white arrow), are shown.

The eruption started on October 24th, with the deposition of a thin bed of fine ash to the east^11^. This short opening event heralded the main explosive phase, which started around noon of October 24th with the formation of a 25 km high eruptive column that, favored by stratospheric winds, caused the propagation of a south-eastwardly dispersed volcanic plume. The Roman towns and villages around Somma-Vesuvius and along the plume dispersal axis were covered by pumice lapilli and ash with thickness up to 3 m at Pompeii^9^, which caused roof collapse of several houses. After a few hours, the plume became unstable and partially collapsed, generating small volume PDCs that hit the slopes of the volcano and buried the town of Herculaneum^9,10^ (Fig. 1a,b). The main explosive phase ended in the morning of October 25th, with the eruption resuming after a few hours with a high column that suddenly collapsed, generating the most destructive PDC of the whole eruption (the EU4 unit), causing injuries up to 20 km south from the volcano^10,11^. The EU4 unit invaded Pompeii (about 10 km from the vent), causing the death of people not yet escaped from the town. Pompeii is a particularly important site for evaluating the impact of an eruption on human beings, because during the eighteenth century excavations archaeologists found a way of producing plaster casts of the victims, giving clues on the effect that the PDCs had on people^12^.

Our survey at the archaeological excavation of Pompeii allowed the visit of the site of Casa di Stabianus (Regio I, insula 22), where in the perimeter of a house some corpses lay embedded by the sediment that formed after the passage of the flow that deposited the EU4 unit. The EU4 deposit rests on top of the fallout pumice bed of the main explosive phase, meaning that the PDC entered the house through the openings and the collapsed roof, and engulfed people that were resting in the house in the time interval between the two main phases of the eruption^12,13^. The deposit consists of a 0.23 m thick bed with internal stratification (Fig. 1c), showing tractional structures such as sand waves. These are the typical features of deposits formed from a dilute current, where ash particles are sustained by turbulence until they settle out of suspension and into a bed load^14–16^. The deposit was formed by continuous aggradation, i.e. by the stacking up of one ash lamina over the other, during the time-integrated passage of the current.

Some preliminary indication of the impact that the PDCs had on human beings comes plaster casts of the bodies that lay embedded in the ash layer (Fig. 1d). They show intact bodies without evidence of any traumatic sign^12^ and suggest that the current did not possess a high dynamic pressure (i.e. high dynamic pressure). Furthermore, clothes are preserved and show that the original texture was not burnt by the passage of the PDC, indicating a temperature below the clothes decomposition, which ranges between 130 and 150 °C for silk and wool, respectively^17^.

### The impact parameters of the PDC at Pompeii

The approach we used to reconstruct of the impact parameters is described in the method section, which includes a description of the equations, from (3) to (14), up on which the model is based. The main data used as input in the model are reported in Table 2. Here the results of flow dynamic pressure, temperature and duration, as representing the main impacts, are illustrated.

**Table 2:** Pompeii deposit data used as input in the model.

*H*_*dep*_ (m)	*ρ*_*dep*_ (kg/m^3^)	*d*_*juv*_ (mm)	*ρ*_*juv*_ (kg/m^3^)	*C*_*d juv*_	*d*_*xx*_ (mm)	*ρ*_*xx*_ (kg/m^3^)	*C*_*d xx*_
0.23	1900	0.40	2200	1.73	0.19	3280	1.39

#### Flow dynamic pressure

**Figure 2:**
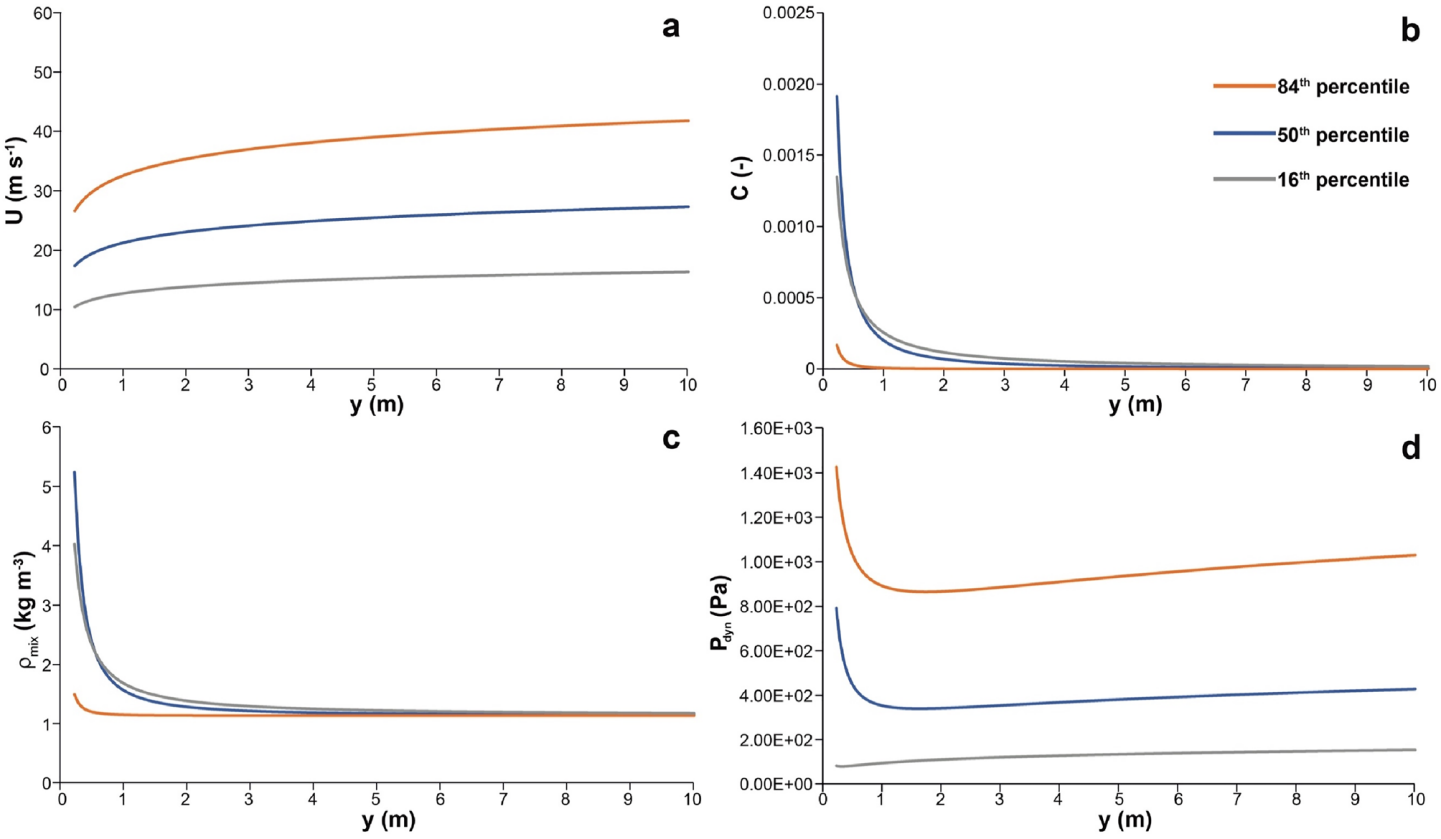
Profiles of the impact parameters representing the flow dynamic pressure. The curves refer to the minimum (16th percentile), the average (50th percentile) and the maximum (84th percentile) of the probabilistic model solution. (**a**)—Velocity profiles. (**b**)—Particle volumetric concentration profiles. (**c**)—Density profiles. (**d**)—Dynamic pressure profiles. British Geological Survey (UKRI) 2021.

In order to illustrate how flow strength varies as a function of current height at Pompeii, the profiles of particle concentration, density, velocity and dynamic pressure are shown on Fig. 2. Results are presented by means of three curves representing the minimum (16th percentile), the average (50th percentile) and the maximum (84th percentile) solution of the probability density functions that were calculated with the method of Dioguardi and Dellino^18^ (see the method section). Velocity, *U,* while increasing upward in the flow (Fig. 2a), reaches values in the range of a few tens m/s. Concentration, *C,* strongly decreases with height (Fig. 2b), and already in the first few meters is lower than 0.001. The density profile, *ρ*_*mix*_ (Fig. 2c), mimics the trend of the concentration profile, and already in the lower two meters decreases rapidly upward to a value lower than atmosphere, making the upper part of the current buoyant. The dynamic pressure *P*_*dyn*_, which represents the combination of velocity and density, has a maximum in the first few decimeters (Fig. 2d). Higher in the current, dynamic pressure is lower than 1 kPa. With these values, no severe mechanical damages are expected to structures, infrastructure or human bodies.

#### Flow temperature

Flow temperature of the current was calculated by using as input in Eq. (11) (see the method section) the values of density, concentration, temperature and specific heat of the three components of the gas-particle mixture, namely: magmatic gas, air and volcanic particles. The temperature of magmatic gas and of volcanic particles was set to 850 °C*,* which is compatible with the 79 AD eruption composition^19^. Air temperature was set to 18 °C, which is a reasonable value for the Somma-Vesuvius area at sea level in the autumn season^20^. Average density was set to 1700 kg/m^3^ for the volcanic particles, to 0.2 kg/m^3^ for volcanic gas at 850 °C and to 1.2 kg/m^3^ for air at 18 °C*,* respectively. The specific heats were set to 2200 J/kg °C for volcanic gas, 700 J/kg °C for the volcanic particles and 1005 J/kg °C for air. As for particle concentration, an average value of 0.001 was set, obtained by integrating the concentration profile of the average solution over flow height (see Fig. 2b) by means of Eq. (4). The relative concentrations of magmatic gas and air were obtained by means of $\rho_{g} = \rho_{m} C_{m} + \rho_{a} \left( {1 - C_{m} } \right)$ and by using as gas density the value calculated by the system of Eqs. (8) and (9). The concentration values of air and volcanic gas resulted 0.941 and 0.058, respectively. By setting all parameters in *(11)*, a temperature of 115 °C was obtained. Zanella et al.^21^ and Cioni et al.^19^ made measurements on the PDC deposit at Pompeii, which indicated temperatures, at the time of deposition, ranging between 140 and 300 °C, which is consistent with the values obtained in this paper when considering that the temperature in the compacted deposit can be a little higher than that of the dilute gas-particle mixture.

The low temperature that we calculated at Pompeii is due to the much higher content of cold atmosphere air in the current, with respect to the hot magmatic gas. This is attributed to the air entrainment process that characterizes PDCs along runout. It is the sum of the air entrainment that occurs at the turbulent interface between the flow head and atmosphere, which is regulated by the Richardson number of the current $Ri_{0} = \frac{g^{\prime}Hcosa}{{U^{2} }}$ where $g^{\prime} = \frac{{\rho_{mix} - \rho_{a} }}{{\rho_{a} }}$ *g* is the reduced gravity^22^, and of the entrainment due to the ingestion of air occurring upon the impact of the eruptive column with the ground. The latter effect is particularly efficient in diluting magmatic gas with atmosphere air in the vicinity of the volcano, as it has been reported both by experiments^22,23^ and by observation of recent eruptions^24^.

#### Flow duration

Flow duration was calculated by using as input in Eqs. (12) and (13) (Method section) data obtained both directly on the PDC deposit at Pompeii, and by means of laboratory analyses carried out on ash samples. Among input, particle concentration, Rouse number and settling velocity are all functions of the shear flow density, which was calculated in terms of a probability density function with PYFLOW v2.0^25^. As a consequence, the results of flow duration are also expressed in terms of probabilities. The average value of flow duration was about 17 min. This duration is quite long when compared to the couple of minutes considered as a survivable time for people engulfed in a PDC, even at low temperature^2,7^.

Our flow duration represents the time during which the layer thickness was formed by continuous settling of particles out of suspension. It does not take into consideration the waning phase of the current, where sedimentation could have been minimal and not completely recorded in the deposit layer, or any periods of nondeposition through bypassing, or pulses of erosion.

Indeed, the time here calculated is to be considered as a minimum estimation. This flow duration represents, therefore, the phase when the current had a significant load of life-threatening ash.

## Discussion and conclusion

The PDCs of the AD 79 eruption of Somma–Vesuvius show a major difference between proximal and distal areas in terms of impact. In the vicinity of the volcano the main effect was related to dynamic pressure and temperature^19,26^. This conclusion is corroborated by our observations at Herculaneum (Fig. 1a), where the current left a massive layer formed by a highly concentrated and hot flow that was capable of breaking and toppling thick walls and of charring wood (see Fig. 1b). These characteristics are indicative of a highly destructive event that did not permit survival, as discussed by previous authors^27,28^.

The situation in distal locations, such as in Pompeii, 10 km from the volcano, is quite different (Fig. 1a). Here, the thermal and mechanical affects. The thermal and mechanical effects of the dilute PDC drastically diminished there. If we integrate the profile of the average solution of the dynamic pressure over the first 10 m (a typical building height in the Vesuvian area) a value lower than 1 kPa results. According to engineering investigations^2,3^, no damage to walls should be expected with such a flow strength, which is consistent with the fact that at Pompeii the walls of Roman buildings do not show evidence of damage^12,29^ related to the passage of the PDC. Furthermore, the bodies embedded in the ash bed do not show any evidence of bone dislocations or fracture, and the bodies look intact, which is consistent with the low flow strength. Even the clothing, whose textures remain visible throughout the plaster casts, look intact. This is in agreement with the low temperature (115 °C) of the gas-particle mixture calculated in this study.

The average value of flow duration that we calculated is about 17 min, which combined with the concentration of ash particles (about 0.001), was a long enough time to cause death by asphyxia at Pompeii. The recent literature on the subject suggests, in fact, that the exposure to fine ash, even at a low particle concentration, can be survived only for a couple of minutes^7^. The flow duration of PDCs can be shorter or longer than this, depending on the scale of the eruption. There are reports of recent eruptions showing that in the marginal reaches of the current, where the flow duration was only a few minutes, people were able to survive^7^. In other cases, longer flow durations did not permit survival and death was caused by fine-ash inhalation^7,30^. Flow duration is a key factor for assessing the impact of PDCs on human beings, especially in distal areas, where the primary risk to life is asphyxiation, as at Pompeii. We agree with Baxter et al.^7^ that the emergency planning for explosive eruptions should concentrate on the distal parts of PDCs where survival could be likely, and where the primary risk to life is asphyxiation from ash inhalation, rather than thermal or mechanical injury.

For Pompeii, we were able to reconstruct flow duration using a novel method that was applied for the first time in this paper. Our method should be used to infer the probable duration of pyroclastic density currents in future events, with this contributing to hazard assessment of active volcanoes.

## Method

The reconstruction of the impact parameters of PDCs is based on a flow mechanical model that starts with the assumption that the current is velocity and density stratified^15,31,32^. In the stratified multiphase gas-particle current, the basal part is a shear flow that moves attached to the ground and has a density higher than atmosphere. The upper part is buoyant, because particle concentration decreases with height down to a value that, combined with the effect of gas temperature, makes the mixture density lower than the surrounding atmosphere.

The inputs needed, in our model, for the calculation of the impact parameters at Pompeii are reported in Table 2. Some of the input data are obtained directly in the field, such as deposit and lamina thickness*.* Deposit density is obtained by weighing a known volume of deposit. Other data come from laboratory analyses on samples extracted from the deposit. In the laboratory, first, the grain-size distribution is determined, then from each size class a sample of particles per each component (crystal, glass, lithics) is extracted, and density data are obtained on such particle samples by means of pycnometers^33^. Particle shape parameters, which are needed for the calculation of settling velocity, are obtained by image analysis methods^34^.

In a PDC, particles are mainly transported by turbulent suspension and sedimentation is controlled by a balance between flow shear velocity u_*_, which is controlled by fluid turbulence and favors suspension, and particle settling velocity, *w*_*t*_ = *(4gd(ρ*_*s*_ *−ρ*_*mix*_*)/3C*_*d*_*ρ*_*mix*_*)*^*0.5*^, which favors sedimentation, where *g* is gravity acceleration, *d* is particle size and *C*_*d*_ is drag coefficient. The median of the grain-size distribution was used for particle size. The capacity of a current to transport particles in suspension is quantified by the Rouse number^35^ $P_{n} = \frac{{w_{t} }}{{ku_{*} }}$, where *k* is the Von Karman constant (0.4). During sedimentation, it is assumed that the particles of different composition that form a lamina settle at the same aerodynamic conditions, e.g., with the same terminal velocity^15^. Therefore, by equating the settling velocity of the glass and crystal components in the deposit, and assuming that sedimentation starts when *P*_*n*_ = 2.5, hence when *w*_*t*_ = u_*_, flow shear velocity and density *ρ*_*sf*_ of the shear flow can be calculated after *d*, *ρ*_*s*_ and *C*_*d*_ are measured in the laboratory^36^. These results are then input in a numerical code^18,25^ and the current parameters are reconstructed. The velocity profile follows the equation of a turbulent boundary layer shear flow moving over a rough surface^37^

3 $$ U\left( y \right) = u_{*} \left( {\frac{1}{k}ln\frac{y}{{k_{s} }} + 8.5} \right) $$

where *k*_*s*_ is the substrate roughness (measured in the field as 0.1 m at Pompeii) and *y* is flow height.

4 $$ C\left( y \right) = C_{0} \left( {\frac{{y_{0} }}{{H - y_{0} }}\frac{H - y}{y}} \right)^{{P_{n} }} $$

where *C*_*0*_ is the particle volumetric concentration at the reference height *y*_*0*_ and *H* is the total current thickness. In this work, *y*_*0*_ is taken as the basal lamina thickness, hence *C*_*0*_ is the particle concentration in the lamina (0.7 in this paper). Assuming steady sedimentation, *H* is obtained by the ratio *H*_*dep*_*/C*_*sf*_ where *H*_*dep*_ is deposit thickness and *C*_*sf*_ is the depth-averaged concentration in the basal shear flow, which can be calculated by *ρ*_*sf*_ = *ρ*_*s*_* C*_*sf*_ + *ρ*_*g*_*(1-C*_*sf*_*),* when *ρ*_*sf*_ and *ρ*_*g*_ are known.

The shear flow height and density are obtained by solving the system of (*5*) and (*6*), which is valid for a turbulent current

5 $$ \tau = \left( {\rho_{sf} - \rho_{a} } \right)gsin\alpha H_{sf} $$

6 $$ \tau = \rho_{sf} u_{*}^{2} $$

where $\tau$ is the shear-driving stress of the flow moving down an inclined slope of angle $\alpha$, in our case 3.2°, measured in the field.

The density profile, which is a function of concentration, particle density and gas density, is:

7 $$ \rho_{mix} \left( y \right) = \rho_{g} + C_{0} \left( {\frac{{y_{0} }}{{H - y_{0} }}\frac{H - y}{y}} \right)^{{P_{n} }} \left( {\rho_{s} - \rho_{g} } \right) $$

Gas density and Rouse number are obtained by solving numerically the following system:

8 $$ \rho_{a} \left( y \right) = \rho_{g} + C_{0} \left( {\frac{{y_{0} }}{{H - y_{0} }}\frac{{H - H_{sf} }}{{H_{sf} }}} \right)^{{P_{n} }} \left( {\rho_{s} - \rho_{g} } \right) $$

9 $$ \rho_{sf} = \frac{1}{{H_{sf} - y_{0} }}\mathop \smallint \limits_{{y_{0} }}^{{H_{sf} }} \left( {\rho_{g} + C_{0} \left( {\frac{{y_{0} }}{{H - y_{0} }}\frac{H - y}{y}} \right)^{{P_{n} }} \left( {\rho_{s} - \rho_{g} } \right)} \right)dy $$

Equation (8) states that atmospheric density, $\rho_{a}$, is reached at the top of the shear flow, *H*_*sf*_, and Eq. (9) states that the average density of the shear flow, $\rho_{sf}$ refers to the part of the flow that goes from the reference level, *y*_*0*_, to the shear flow top height, *H*_*sf*_.

By combining the velocity and density profiles, the dynamic pressure profile is finally obtained

10 $$ P_{dyn} \left( y \right) = 0.5U^{2} \left( y \right)\rho_{mix} \left( y \right). $$

The profiles of the flow parameters are expressed in terms of a probability density function that depends on the variance of particle characteristics. The model has been validated by experiments^3^ and already applied to other eruptions^15,33^.

The temperature of a PDC is quantified as the weighted average between the relative proportions of the three components that make up the gas-particle mixture, namely the volcanic gas and solid particles that issue from the crater, plus the atmospheric air that is entrained by the current during its spreading. The temperature of the mixture can be approximated by

11 $$ T_{mix} = \frac{{\rho_{g} T_{g} C_{g} Cp_{g} + \rho_{s} T_{s} C_{s} Cp_{s} + \rho_{a} T_{a} C_{a} Cp_{a} }}{{\rho_{g} C_{g} Cp_{g} + \rho_{s} C_{s} Cp_{s} + \rho_{a} C_{a} Cp_{a} }} $$

where *T* and *Cp* are the temperature and specific heat (at constant pressure), respectively. The subscripts *g*, *s* and *a* stand for gas, solid particle and air, respectively.

Concerning flow duration, in a PDC, sedimentation occurs at a rate $$S_{r} $$ that represents the mass of particles settling over a unit area in the unit time. Deposit thickness grows by aggradation of ash laminae during the time-integrated passage of the current. The aggradation rate $$A_{r}$$, which is the rate at which deposit thickness grows, is equal to the sedimentation rate divided by deposit density, $$\rho_{dep}$$.

The total time of aggradation, *t*, which is a proxy of flow duration, is equal to deposit thickness divided by the aggradation rate, *A*_*r*_, which is represented by the ratio of deposit density and sedimentation rate:

12 $$ t = \frac{{H_{dep} }}{{A_{r} }} $$

Deposit density and thickness are measured in the field, consequently the only missing quantity for the calculation of flow duration is the sedimentation rate.

Dellino et al. ^38^, recently proposed a model for the calculation of the sedimentation rate

13 $$ S_{r} = \left( {\mathop \sum \limits_{i}^{n} \rho_{{s_{i} }} w_{{t_{i} }} \left( {\frac{{\frac{{\phi_{i} /\rho_{{s_{i} }} }}{{\mathop \sum \nolimits_{i = 1}^{n} \phi_{i} /\rho_{{s_{i} }} }}*C_{tot} }}{{\left( {\left( {10.065*\frac{{P_{ni} }}{{P_{n}^{*} }}} \right) + 0.1579} \right)}}*0,7 + \frac{{\frac{{\phi_{i + 1} /\rho_{{s_{i} + 1}} }}{{\mathop \sum \nolimits_{i = 1}^{n} \phi_{i + 1} /\rho_{{s_{i + 1} }} }}*C_{tot} }}{{\left( {\left( {10.065*\frac{{P_{ni} }}{{P_{n}^{*} }}} \right) + 0.1579} \right)}}*0.3} \right)} \right) - 0.01. $$

with the subscript *i* referring to the *i*_*th*_ particle-size class and *n* being the number of size classes of the grain-size distribution of the sediment, with $\phi_{i}$,${ }\rho_{{s_{i } }}$ and $P_{ni}$ being the weight percent, the density and the Rouse number of the ith grain-size fraction, respectively. *P*_n_* = *P*_*navg*_*/P*_*nsusp*_ is the normalized Rouse number of the current, i.e. the ratio between the average Rouse number of the solid material in the current and the Rouse number at maximum suspension capacity. The model considers the contribution of each size class of particles to the sedimentation, and not the average grain size, because the solid load constituting a suspension current, especially in the case of PDCs, is made up of a mixture of different components (lithics, glassy fragments and crystals) with different size, density and shape, thus different terminal velocity. The average Rouse number of the solid material in the current is calculated as the average of the particulate mixture,

14 $$ P_{{n_{avg} }} = \mathop \sum \limits_{i = 1}^{n} P_{ni} C_{i} /C $$

When *P*_n_* is higher than 1, a current has a particle volumetric concentration in excess of its maximum capacity, e.g. it is over-saturated of particles, which favours sedimentation. When it is lower than 1, a current has a particle volumetric concentration lower than its maximum capacity, e.g. it is under-saturated, and could potentially include additional sediment in suspension by erosion from the substrate. For a specific discussion see Dellino et al.^38^.
